# Laser Sintering by Spot and Linear Optics for Inkjet-Printed Thin-Film Conductive Silver Patterns with the Focus on Ink-Sets and Process Parameters

**DOI:** 10.3390/polym16202896

**Published:** 2024-10-14

**Authors:** Dana Mitra, Kalyan Yoti Mitra, Georg Buchecker, Alexander Görk, Maxim Mousto, Thomas Franzl, Ralf Zichner

**Affiliations:** 1 Fraunhofer Institute for Electronic Nano Systems ENAS, 09126 Chemnitz, Germany; dana.mitra@enas.fraunhofer.de (D.M.); georg.buchecker@enas.fraunhofer.de (G.B.); ralf.zichner@enas.fraunhofer.de (R.Z.); 2 Hamamatsu Photonics Deutschland GmbH, 82211 Herrsching am Ammersee, Germany; alexander.goerk@hamamatsu.eu (A.G.); maxim.mousto@hamamatsu.eu (M.M.); tfranzl@hamamatsu.de (T.F.)

**Keywords:** inkjet printing technology, laser sintering, nanoparticle silver ink, flexible electronics

## Abstract

The implementation of the laser sintering for inkjet-printed nanoparticles and metal organic decomposition (MOD) inks on a flexible polymeric film has been analyzed in detail. A novel approach by implementing, next to a commonly 3.2 mm diameter spot laser optic, a line laser optic with a laser beam area of 2 mm × 80 mm, demonstrates the high potential of selective laser sintering to proceed towards a fast and efficient sintering methodology in printed electronics. In this work, a multiplicity of laser parameters, primary the laser speed and the laser power, have been altered systematically to identify an optimal process window for each ink and to convert the dried and non-conductive patterns into conductive and functional silver structures. For each ink, as well as for the two laser optics, a suitable laser parameter set has been found, where a conductivity without any damage to the substrate or silver layer could be achieved. In doing so, the margin of the laser speed for both optics is ranging in between 50 mm/s and 100 mm/s, which is compatible with common inkjet printing speeds and facilitates an in-line laser sintering approach. Considering the laser power, the typical parameter range for the spot laser lays in between 10 W and 50 W, whereas for the line optics the full laser power of 200 W had to be applied. One of the nanoparticle silver inks exhibits, especially for the line laser optic, a conductivity of up to 2.22 × 10^7^ S‧m^−1^, corresponding to 36% of bulk silver within a few seconds of sintering duration. Both laser sintering approaches together present a remarkable facility to use the laser either as a digital tool for sintering of defined areas by means of a spot beam or to efficiently sinter larger areas by means of a line beam. With this, the utilization of a laser sintering methodology was successfully validated as a promising approach for converting a variety of inkjet-printed silver patterns on a flexible polymeric substrate into functionalized conductive silver layers for applications in the field of printed electronics.

## 1. Introduction

The inkjet technology is a highly recognized and established printing and manufacturing technology in general [1,2,3] and for applications in printed functionalities [4,5]. Printed electronic devices, manufactured especially via Inkjet technology, are a huge developing field, with examples in printed TFTs [6], antennas [7,8], capacitors [9], sensors [10], or inductors [11], just to mention a few.

Today’s printed electronic applications are integrated into a multiplicity of objects in various environments [12,13,14]. This demands a high adaptability of the electronics to be either fabricated on any thin and flexible substrate, which is subsequently attached to the final application or object, or the device that is directly printed onto any planar or non-planar three-dimensional object. In both cases, the manufacturing process needs to be adapted to the respective background material, which means not only the printing but also the post-treatment needs to be suitable for diverse substrates, especially when it comes to materials with low thermal stability [6,7,8,9,10,11,12,13,14]. Furthermore, an adaptable and distinct selective sintering process is advisable to enable integrations in sheet-to-sheet (S2S), roll-to-roll (R2R), as well as free-form-platform manufacturing processes. One promising realization is the combination of digital and contactless inkjet printing with selective laser sintering. The inkjet printing of conducting material together with thermal sintering is a recognized and well established methodology [15,16,17]. Also, novel sintering methods by means of infrared (IR) radiation [18], microwaves [19,20], plasma [19], and intense pulsed light (IPL) [19,20,21,22,23] are important research topics and fundamentally discussed. This also includes the methodology of the laser sintering [19,20,21,24,25,26,27,28,29,30,31,32,33,34], which is in detail demonstrated in various publications.

Within these publications, the laser optics was always a spot or elliptical beam with a diameter of only several millimeters. Therefore, the sintering was only carried out for printed lines within a line width of a few µm or mm for basic investigations on, e.g., the pulse overlapping [30], thermal conductivity [24], or defect analysis with the printed layer and the substrate [25]. As demonstrated in the research of Yang et. al. [27], the sintering of an antenna pattern of approx. 37 mm × 63 mm was performed line by line with 27 min, as the laser sintering process was performed with 10 and 50 mm/s. During these various publications, laser sintering was shown using continuous wave (CW) fiber, pulsed nano- or picosecond lasers for either a nanoparticle or a MOD ink, and the focus was dedicated towards a specific and partly also analytic survey of the impact of, e.g., laser speed, laser power/intensity, spot gap, or pulse overlap.

However, the novelty in our work is the demonstration of large scale laser sintering for a broader range of inks on a flexible substrate towards a fast and efficient R2R-applicable sintering process. For the first time, we demonstrate with this publication how laser sintering can be approached with dedicated Hamamatsu laser equipment with two differing laser optics (a laser spot optic with a 3.2 mm beam diameter and a line optic with a 2 mm × 80 mm laser beam geometry) and implement it to fully sinter an inkjet printed MOD ink and a couple of nanoparticle silver inks. We focus on standardized inkjet printed silver layers with a thickness that was not tuned for the laser sintering process; instead, the parameters for the printing process were fixed for the individual silver inks.

Within our research, we have also shown how the thickness of the layer and the topology can influence the sheet resistance of the printed conductive silver layer. When compared in literature, the highest conductivity through the laser sintering process was reported to be 26% [26], whereas within this research paper we demonstrate the highest ever reached conductivity from inkjet printed silver ink, i.e., 36% (conductivity of 2.22 × 10^7^ S·m^−1^) using a CW line laser from Hamamatsu, which is in itself a big technological milestone. Last but not least, in the literature there has always been a missing key element for industrialization, which we have also covered with our experimentation, i.e., scale-up and processibility for patterns vs. sintering processes. We have shown that by using spot and line optic lasers, one can focus on the sintering process, in-line with the inkjet printing step, and also have versatile flexibility towards printing numerous varieties of printed pattern shapes, sizes, and designs to achieve the required device specification.

## 2. Materials and Experimental Setup

**Figure 1:**
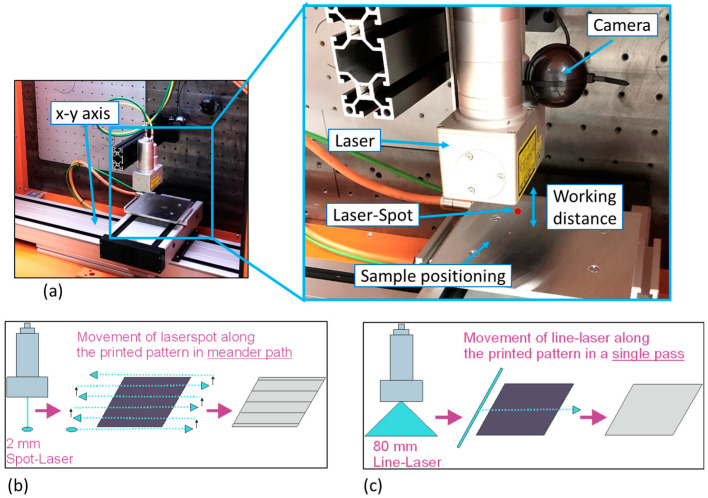
Overview of the laser-sintering setup and procedure: (**a**) photographs of the installed laser module and X-Y-table graphical illustration for the laser-sintering routine with (**b**) a spot-laser and (**c**) a line-laser.

The inks used for the experiments were two NP “nanoparticle silver” inks (Ag-NP1 and Ag-NP2) as well as one MOD “metal-organic decomposition” or particle-free ink (Ag-MOD). A variety of different sized patterns (5 × 5 mm^2^, 3 × 3 mm^2^, and single lines with 1–3 pixel width) were inkjet-printed (Dimatix Materials Printer DMP-2831 from Fujifilm Dimatix Inc., Santa Clara, CA, USA) with a resolution of 1270 dpi on 125 µm thick polyethylene naphthalate (PEN) sheets and only dried before the printed non-conductive patterns were utilized for sintering tests. The samples were individually placed on top of an X-Y axis table (Figure 1a) to execute a huge variety of laser sintering procedures, in which the laser power [*p* in W] and the sintering speed [*v* in mm/s] were systematically investigated. The laser power itself is controlled by the electrical power input to the laser diodes. In our experiments, the 200W T-SMILS L15570-311 (including absolute temperature monitoring) with the integrated laser module SPOLD L13920-611J from Hamamatsu Photonics Deutschland GmbH (Herrsching am Ammersee, Germany) has been used. Additionally, the laser was either equipped with a spot- (irradiation unit A12254-41B) or a line-optics (irradiation unit A13933-13W). The spot-optics has a light condensing spot diameter of 3.2 mm and a working distance of approximately 90 mm between the laser irradiation unit and the substrate. The line-optics had an oval or line-shaped beam size with a width of 80 mm and a working distance of approximately 143 mm. Both the lasers can be operated with a maximum incident power of 200 W. For the line-laser setup (Figure 1c), a single sintering path was enough to cover the whole area of the printed patterns, whereas for the spot-laser (Figure 1b), the laser had to run in several iterative paths with a defined pitch/shift along the printed pattern to homogeneously cover and sinter the complete area. After a huge variety of laser sintering tests, parameter definition, and adaptation, the most promising results were transferred to a large scale setup, where patterns up to 20 mm × 20 mm were laser sintered with the spot as well as the line optics.

## 3. Results and Discussion

### 3.1. Parameter Range and Characteristics for the Spot-Laser Sintering

For all three inks, a huge series of experiments were executed to find out the optimal parameter range to achieve the lowest resistance without damaging the printed patterns or substrate. Each ink revealed its own ideal working range, which was more or less adaptable to changes in either laser power or sintering speed. The laser parameters were mainly chosen to facilitate fast and efficient sintering but also in accordance with common inkjet printing speeds, which are typically in the range of 25 mm/s to 100 mm/s for manufacturing thin-film electronics, to ensure a stable and high printing quality. For each ink, a different optimal set of laser parameters was found, reveling more or less flexibility towards variations in the laser power and sintering speed.

**Figure 2:**
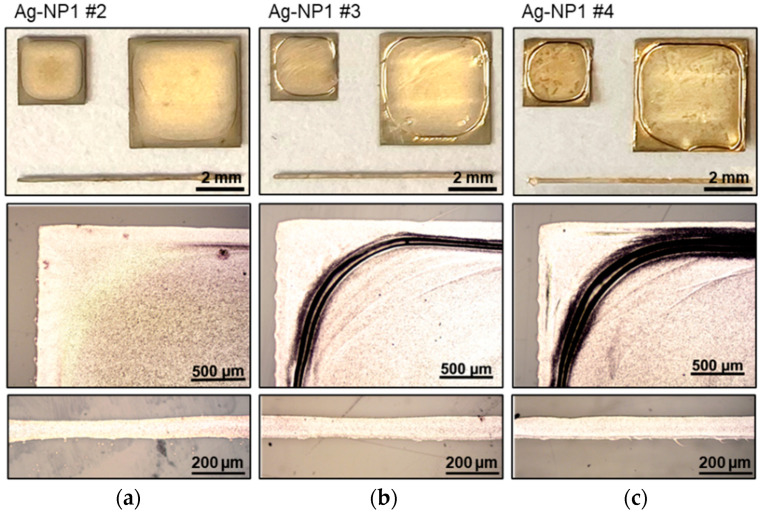
Photographs and microscopic images of the ink Ag-NP1 that is printed and sintered with laser parameters (power and sintering speed): (**a**) 8 W and 50 mm/s; (**b**) 10 W and 50 mm/s, and (**c**) 12 W and 50 mm/s.

The first silver ink, Ag-NP1, obtained a complete electrical conductivity at laser speeds of 50 mm/s. Operating faster or slower laser speeds leads to either insufficient (not homogeneous conductivity throughout the printed pattern) or destructive sintering (cracks or other defects), irrespective of versatile laser power adjustments. In Table 1, it can be seen that at a constant laser speed of 50 mm/s, an increase in laser power from 6 W to 8 W induces a change in electrical resistance from several Megaohms (not conductive) to approximately 1 Ω, demonstrating the high impact from just a 2 W power change. Increasing the laser power stepwise by 2 W decreases the electrical resistance gradually but also induces damage growth (Figure 2b,c) at the edges. A typical characteristic of this ink is a ring contoured material accumulation around the periphery of the printed pattern, which turns into an undesired working point for cracks and delamination during the laser sintering with higher power. As already mentioned, an increase in laser speed and power or vice-versa, a decrease in laser speed and power, results in either unsatisfactory electrical conductivity or unchanged defect appearance. Reducing these effects will only be managed by a change in the printing strategy, i.e., by implementing thinner layers or even slower drying procedures, which in turn will increase the layer homogeneity.

**Figure 3:**
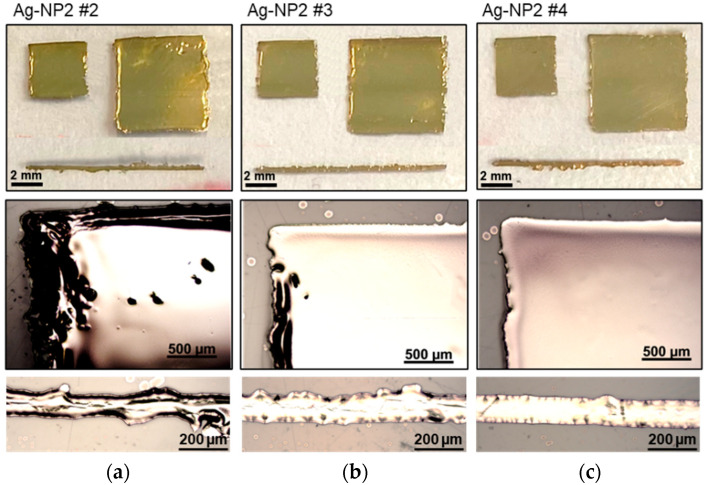
Photographs and microscopic images of the ink Ag-NP2 that is printed and sintered with laser parameters (power and sintering speed): (**a**) 26 W and 50 mm/s; (**b**) 26 W and 75 mm/s, and (**c**) 26 W and 100 mm/s.

The second silver ink Ag-NP2 revealed a comparable parameter window with higher flexibility in the laser power adjustments, as shown in Figure 3 and Table 2. At a laser power of 26 W, the laser speed needs to be above 75 mm/s to reduce and prevent damage to the silver layers. Furthermore, it was found that laser power below 26 W leads again to inefficient sintering and electrical characteristics. Thus, the optimal sintering process window was found within a sintering speed of 100 mm/s and a laser power of 26 W (Figure 3c). A further increase in the laser power led to a slight drop in electrical resistance. On the contrary, a laser power of 32 W should not be exceeded due to the emerging of unforced cracks and partially peeling-off of the silver layer.

For both the nanoparticle silver inks, the optimal process window was found, where no or minor defects with high conductivity could be achieved. It was demonstrated that this process window is within a quite narrow optimal parameter range that is balanced between the laser power and sintering speed.

**Figure 4:**
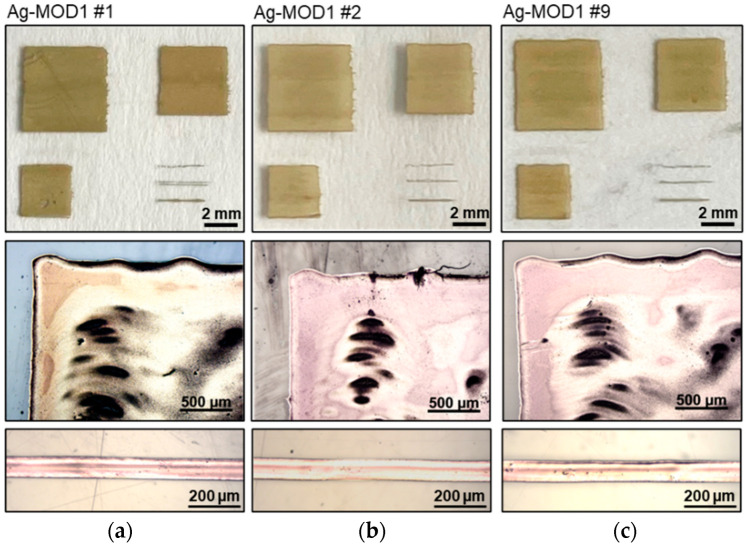
Photographs and microscopic images of the ink Ag-MOD that is printed and sintered with laser parameters (power and sintering speed): (**a**) 18 W and 50 mm/s; (**b**) 20 W and 75 mm/s; and (**c**) 20 W and 100 mm/s.

The third experimental run highlights the laser sintering of a MOD silver ink (Ag-MOD). This type of ink was exemplarily chosen to not only demonstrate the challenge of laser sintering of a particle free ink but also the potential it might facilitate, i.e., flawless high resolution inkjet printing. As it can be seen in Table 3, the ink had a broad parameter spectrum, ranging from 18 W to 40 W at sintering speeds of 50 mm/s to 100 mm/s, and constantly attained a sheet resistance of approximately 0.11 Ω/sq. to 0.15 Ω/sq., with least standard deviations of 0.01 Ω/sq. to 0.05 Ω/sq., allowing a high flexibility of processing speeds according to, e.g., fixed printing speeds. Having a closer look at the microscopic images in Figure 4a–c, small defects can be detected. Irrespective of the change in the laser power or laser speed, these characteristic damages occur regularly and can mostly be explained by unevenness in the layer thickness caused by drying inhomogeneities.

In the case of the inkjet printing technology, the uneven drying in a silver layer mostly happens due to the impact of the deposition process and the in-situ drying phenomenon. As the ink gets deposited swath-wise (multiple step-wise passes) over a given area, the outer edges of the ink layer start to dry relatively faster compared to the central location, and along with this, the upper region of the printed layer starts to develop higher surface tension intermediate zones, ultimately leading to a material pull towards the upper edge. Here, this evaporation-driven material transport phenomenon is majorly aggravated due to the high solvent content in the inkjet silver ink and also the slow laboratory-based inkjet printing process, which is limited by the limited number of printing nozzles (up to 16 only). In our case, during the utilization of MOD silver ink, the layer uniformity is found to be very important, as the sintering process is mainly triggered by the absorption of the laser energy and the execution of the thermal decomposition reaction, converting the silver salt complex to silver nanoparticles that furthermore develop a specific microstructure.

Both sintering parameters were systematically studied, but decreasing the power at a defined speed or increasing the speed at a defined power to reduce or eliminate these defects resulted in insufficient sintering and/or poor conductivity. The investigated MOD ink disclosed a defined energy input, which had to be introduced to trigger the comparable complex decomposition and conversion of the particle free ink into the silver compound. However, this specific energy could be introduced to the printed layers by either lower power at slower speeds, or higher power at higher speeds, facilitating a high adaptability towards a predefined manufacturing process. Reduction, or in the best case, eliminating these microscopic irregularities, can be part of further investigations.

### 3.2. Parameter Range and Characteristics for the Line-Laser Sintering

**Figure 5:**
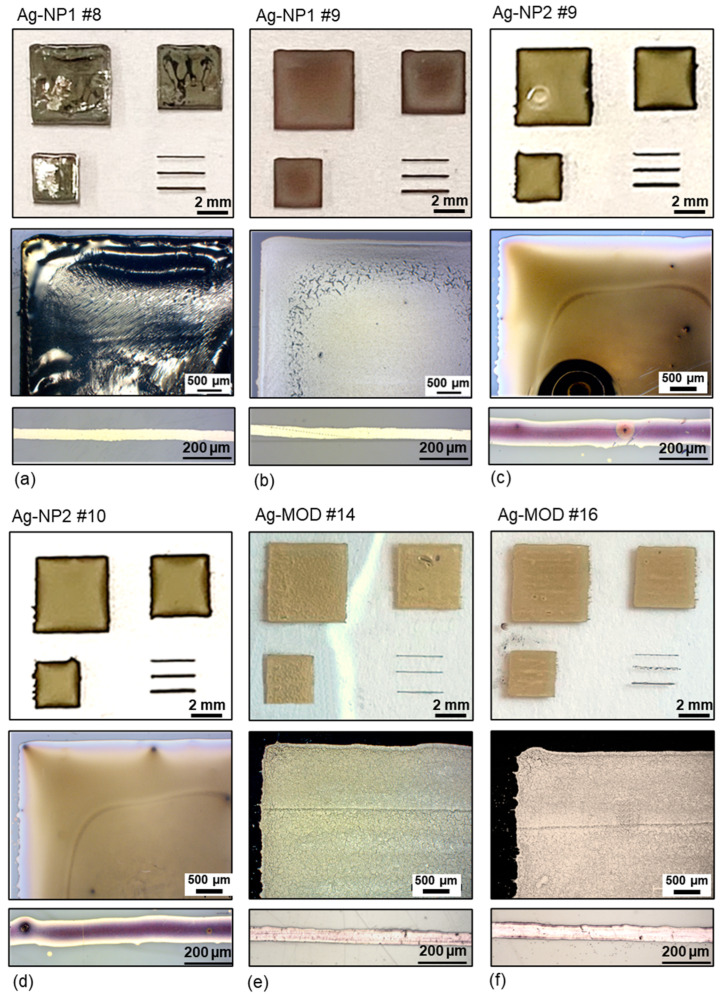
Photographs and microscopic images of all three printed inks sintered with the line laser at 200 W with corresponding ink type and sintering speed of: (**a**) Ag-NP1 #8 at 10 mm/s; (**b**) Ag-NP1 #9 at 20 mm/s; (**c**) Ag-NP2 #9 at 40 mm/s; (**d**) Ag-NP2 #10 at 50 mm/s; (**e**) Ag-MOD #14 at 10 mm/s; and (**f**) Ag-MOD #16 at 20 mm/s.

Compared to the spot laser (3.2 mm beam diameter), the line laser optic widens the laser beam into a 2 mm by 80 mm broad line (Figure 2c). Hence, the laser power is equally distributed over the area, and it is expected to work with much higher laser power. A few preliminary tests confirmed that for all three inks, the highest laser power output of 200 W had to be applied to realize conductive silver patterns. In Figure 5, photos and microscopic images show exemplarily the results of the line-laser sintering.

**Figure 6:**
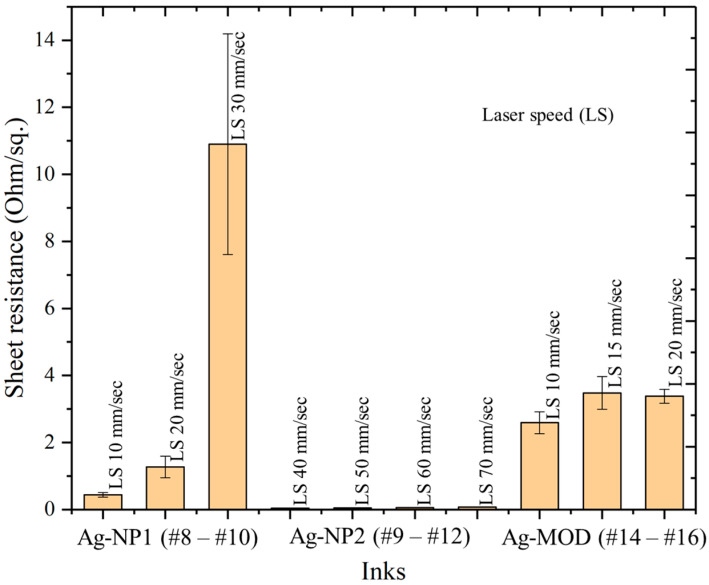
Graph showing parameter set for the line laser sintering and results of measured sheet resistance of Ag-NP1, Ag-NP2, and Ag-MOD.

The deciding parameter was to find the suitable sintering speed where a low sheet resistance without or minimal defects could be achieved. The results of the sheet resistance are exemplarily given in Figure 6. The outcome is a comparable smaller parameter flexibility for the first nanoparticle ink Ag-NP1, as well as for the MOD ink Ag-MOD. For the Ag-NP1, a sheet resistance below 1 Ω/sq. could only be achieved at a low speed of 10 mm/s, but unlikely with burning defects (Figure 5a).

Whereas the step to 20 mm/s could reduce the burning to fine cracks at the edge, the sheet resistance ultimately increased to approximately 1.27 Ω/sq. with a higher standard deviation. On the contrary, the second nanoparticle ink, Ag-NP2, demonstrated that with a sintering speed of 50 mm/s up to 70 mm/s, highly conductive silver patterns below 0.1 Ω/sq. could be achieved without any damage (Figure 5d). On the contrary, for speeds lower than 50 mm/s, some burning defects could appear (Figure 5c). Increasing the speed by more than 70 mm/s, the sintering that occurred was found to be not complete, and the resistance remained increasingly high.

Considering the MOD ink, the laser sintering with a line optic only achieved a sheet resistance down to 2.59 ± 0.33 Ω/sq. with the highest possible parameters of 200 W and 10 mm/s. Reducing the sintering speed to 5 mm/s led to burning of the printed pattern. In this case, it is expected that the laser power needs to be higher so that the sintering speed could be increased and a higher energy sintering at faster speed is enabled. It can be assumed that a higher energy for a shorter time will improve the sintering effect as compared to a lower energy for a longer duration. Also, a double sintering pass with various setups was tested, which only resulted in damaged patterns and substrates. For this specific ink, a laser power of more than 200 W is therefore required for future testing and process development.

**Figure 7:**
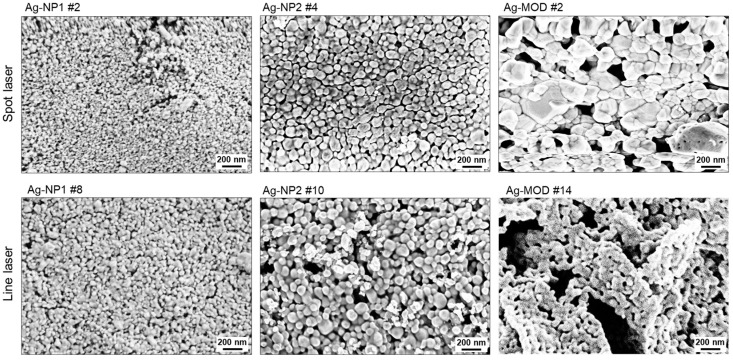
Scanning electron microscopic (SEM) images of selected images for the spot- and the line laser sintering for the three inks, respectively.

In Figure 7, SEM images reveal a deeper insight into the micro- and nanostructure of the laser sintered silver layers. The nanostructure of both nanoparticle silver inks is comparable, disclosing a fine cluster of interconnected nanoparticles in the nanometer range. However, the particle size of the ink, Ag-NP2, appears marginally bigger. Compared to this, the MOD ink discloses larger metallic clusters, which results from the chemistry of the ink itself, which does not contain single nanoparticles but a silver salt complex, reducing to nanoparticles and grain growth induced by the laser energy. Therefore, the metallic microstructure forms larger agglomerates.

Theoretically, we can interpret that due to the chemistry of nanoparticle-based inks, after the sintering process is accomplished, the adhesion of the silver entities to the polymeric substrate is far better due to the presence of organic binders and stabilizers. In contrast to this, silver MOD inks possess simplified chemistry, which results in a thermal/chemical decomposition reaction during the sintering process. Thereby, reducing the silver salt complex to silver nanoparticles that adhere to the substrate uniquely without any stabilizers. So, we can assume that the adhesion and flexibility are not up to the best limits when compared to the nanoparticle-based inks.

### 3.3. Implementation of the Optimized Parameters for Large Scale/Rapid Laser Sintering

For both nanoparticle silver inks, a layout composed of a variety of different sized sample patterns was designed to verify the laser sintering as a suitable tool for printed electronics. The aim was to sinter these sets of differing patterns with the same optimized laser parameters with no or least amount of defects, especially while comparing printed larger, full sized patterns with thin printed lines. The sample patterns in this study were composed of 400 mm^2^, 100 mm^2^, and 25 mm^2^ squares, as well as 200 µm and 500 µm wide lines. The silver ink AG-NP1 was chosen to be sintered with the spot and the line optic, and as a comparison, the second silver ink Ag-NP2 was sintered exemplarily with the line optics. The printing parameters were kept constant, but the drying parameters were slightly adjusted (60 °C for 30 min), which resulted in enhanced uniformity and reduced coffee ring effect or other material topographical inhomogeneities due to a slightly slower drying with respect to larger printed areas. The laser parameters were selected based on the previous outcomes to achieve the lowest sheet resistance with minimal irregularities.

**Figure 8:**
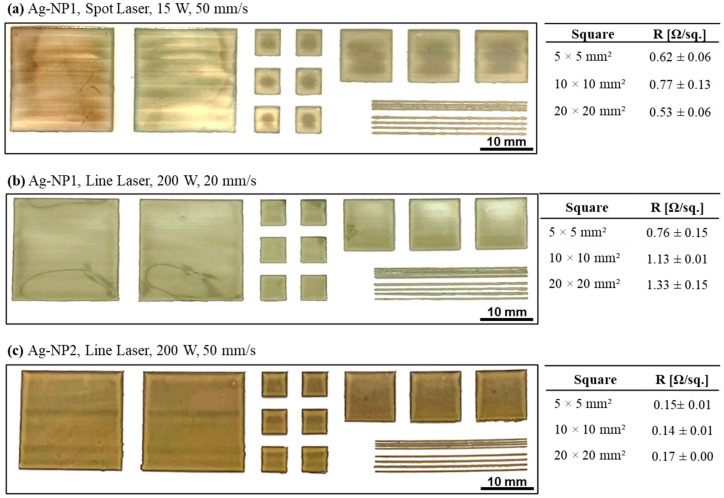
Photographs and corresponding measurements of the sheet resistance for the selected large area laser sintering.

In Figure 8, the results in the form of photographic images and the sheet resistance are summarized. The sheet resistance was taken, and the average was calculated for the individual squares to demonstrate the laser sintering ability towards the functionalization of more individual pattern geometries. The implementation of the spot laser for ink Ag-NP1 (Figure 8a) can be identified by a light line like appearance, i.e., clearly visible for the largest square of 400 mm^2^. The laser spot moves with a pitch of 3 mm line by line along the entire printed pattern. The brightest line structure within the largest square was found to be visible for narrow overlapping areas, where the passing laser spot would meet the already sintered layer location from the previously sintered travel path, leading to a twice sintered narrow overlapping zone. The consequences can be minor in the form of slight irregularities in the sheet resistance but might become critical if such irregularities affect the functionality of the conductive tracks in a negative way or an excessive sintering effect in this region causes defects, such as cracks. It is crucial to align the laser travel path with the highest precision, to keep the overlapping zone as small as possible without being too far apart and ending up with structural discontinuities and therefore compromised resistance or bad conductivities. Additionally, the results of the sheet resistance were found to be about 0.61 ± 0.13 Ω/sq., which is comparably constant for the three square dimensions, and with the most homogeneous drying and adjusted laser parameters, the resistance could be even decreased further compared to the previous results (see Figure 2 and Table 2). In contrast, the line laser sintering revealed an optically very uniform sintering result, but with an average sheet resistance of 0.97 ± 0.27 Ω/sq. considering the three square dimensions, demonstrating a comparatively higher fluctuation among the three square dimensions (Figure 8b). The trend indicates an increasing sheet resistance with increasing pattern size, though the fluctuations within one pattern size are comparatively low (0.01–0.15 Ω/sq.). Based on the previous tests, for the Ag-NP2, only the line laser was applied, as it was expected that the results for both laser optics would be similar for this specific ink. Additionally, minor inhomogeneities in the drying, all the printed and sintered patterns appeared uniform without noticeable defects, and the sheet resistance was found to be about 0.15 ± 0.01 Ω/sq., with high consistency and negligible deviations.

The thickness of the printed and sintered ink layers was determined with the help of surface profilometry and was found to be approximately 0.5 ± 0.1 µm for the ink Ag-NP1, 0.9 ± 0.15 µm for the ink Ag-NP2, and 1.2 ± 0.1 µm for the ink Ag-MOD. Together with the values of the sheet resistance, the average conductivity could be calculated, and an overview of the highest achieved percentage of the bulk silver conductivity for the individual inks and laser sintering methodology is given in Table 4.

To summarize Table 4, it can be said that the silver inks Ag-NP2 and Ag-MOD demonstrated their highest electrical conductivities of 6.17 × 10^6^ S·m^−1^ and 5.49 × 10^6^ S·m^−1^, respectively, leading for both to 10% conductivity of the bulk silver, when the silver patterns were sintered with the spot laser. It is worth mentioning that the spot-laser sintering of the Ag-NP2 silver layers was performed using 26 W and 100 mm/s sintering speed, and that for the Ag-MOD ink, the most optimal results were obtained while applying 20 W and 75 mm/s sintering speed. This demonstrates that two different ink chemistries (nanoparticles vs. MOD) result in a difference in the laser energy absorptions and the energy utilization within the ink layer and demand the implementation of two different process parameters for establishing the specific sintering methodology.

In contrast to this, the same cannot be said for the utilization of the line-optics laser. Here, the energy that is absorbed and utilized for the execution of the sintering process was found to be different, as the power was always set constant at 200 W (maximum of the used laser system) and the laser writing speed was varied. For the change in varying laser writing speeds and constant laser power, the nanoparticle inks showed the better results in general. Especially for Ag-NP2, which exhibited an electrical conductivity of 2.22 × 10^7^ S·m^−1^, leading to 36% electrical conductivity of bulk silver. This confirms the fact that the line-optics laser is much more favorable and compatible for sintering nanoparticle-based ink, which can better absorb the laser energy and trigger the sintering process effectively for obtaining electrically conductive microstructures. Compared to this, for the MOD based silver ink, it is interpreted that the potential of sintering the silver pattern is present, but a higher output power for the line optics is demanded to establish the thermal degradation of the silver complex to conductive nanoparticles.

Supplementing to the usage of the laser sintering for complex and bigger sized patterns with numerous variations, the process parameters for sintering were found to be again favorable for the silver ink Ag-NP2 with the line-laser set-up, where an electrical conductivity of 7.41 × 10^6^ S·m^−1^ was achieved when using the optimal process parameter of 200 W and 50 mm/s laser sintering speed. This results definitely foster the outlook for the utilization of line optic lasers for sintering bigger conductive patterns that are inkjet printed with nanoparticle based inks, foremost addressing the scope for industrialization.

## 4. Conclusions

Within this research work, the implementation of the laser sintering for three different silver inks (two nanoparticles and one MOD ink) that are inkjet-printed on a flexible polymeric foil has been analyzed in detail. For each ink, a multiplicity of laser parameters, explicitly the laser speed and the laser power, have been adapted systematically to evaluate the optimal process window for each ink, where the highest conductivity without any damage to the substrate or silver layer could be achieved. Furthermore, the laser optics implemented here have been either a spot type or a line type with 3.2 mm and 2 mm × 80 mm sized dimensions, respectively. For all these three inks, a suitable laser parameter set has been found for the spot as well as for the line laser optics to convert the dried non-conductive silver patterns into conductive structures. Typical laser speeds for the spot optics were found to be best between 50 mm/s and 100 mm/s, which matches the typical printing speed of R2R inkjet printing. The laser power was adapted in the range of 10 W to a maximum of 30 W to achieve the lowest resistivity without damaging the silver layers. Considering the line optics, the laser power was for all three inks kept constant at 200 W, and the laser sintering speed was adjusted to 20 mm/s to 50 mm/s, which is slightly slower than the spot optic.

The achieved conductivities range between 3% and 10% for the spot optic and 0.5% and 36% for the line optics, considering the defect free sintering. Higher conductivities could be achieved only by compromising on minor defects, such as small cracks or peeling off from the substrate.

With this, it was successfully demonstrated that not only the laser sintering can be deployed for a variety of inkjet-printed silver patterns on a flexible polymeric substrate, but also can be a validated upscaling process tool for rapid and efficient laser sintering. Hence, the laser sintering by means of line optics reveals high potential and scope for an efficient and damage-free functionalization of printed patterns and their application in flexible printed electronics. The future scope of this work is to apply the obtained results on one hand to a real inline printing and laser sintering approach as well as to extend the laser sintering approach to challenging substrates, such as composite materials and textiles.

## Figures and Tables

**Table 1:** Parameter set for the spot laser sintering and results of measured sheet resistance of Ag-NP1.

Ag-NP1			
#	Power [W]	Speed [mm/s]	R [Ω/sq.]
1	6	50	MΩ
2	8	50	1.09 ± 0.04
3	10	50	0.62 ± 0.00
4	12	50	0.34 ± 0.08
5	14	50	0.27 ± 0.03
6	16	50	0.21 ± 0.01
7	18	50	0.18 ± 0.02
8	20	50	Full damage

**Table 2:** Parameter set for the spot laser sintering and results of measured sheet resistance of Ag-NP2.

Ag-NP2			
#	Power [W]	Speed [mm/s]	R [Ω/sq.]
1	26	10	Full damage
2	26	50	0.13 ± 0.01
3	26	75	0.17 ± 0.02
4	26	100	0.18 ± 0.00
5	28	100	0.14 ± 0.00
6	30	100	0.15 ± 0.01
7	32	100	0.12 ± 0.02
8	34	100	Full damage

**Table 3:** Parameter set for the spot laser sintering and results of measured sheet resistance of Ag-MOD.

Ag-MOD			
#	Power [W]	Speed [mm/s]	R [Ω/sq.]
1	18	50	0.14 ± 0.04
2	20	75	0.14 ± 0.05
3	28	75	0.11 ± 0.00
4	30	75	0.12 ± 0.01
5	32	75	0.12 ± 0.01
6	34	75	0.11 ± 0.00
7	36	75	0.14 ± 0.03
8	38	75	0.14 ± 0.02
9	20	100	0.15 ± 0.00
10	22	100	0.15 ± 0.03

**Table 4:** Overview of the best achieved conductivities for laser sintered silver layers is shown according to the laser optics and conducted experiments.

Spot Laser			
**Ink and Sample**	**Average Resistivity [Ω‧m]**	**Average Conductivity [S‧m^−1^]**	**Average Percent of Bulk Ag**
Ag-NP1 #2	5.47 × 10^−7^	1.83 × 10^6^	3%
Ag-NP2 #4	1.62 × 10^−7^	6.17 × 10^6^	10%
Ag-MOD #2	1.68 × 10^−7^	5.49 × 10^6^	10%
**Line laser**			
Ag-NP1 #2	6.35 × 10^−7^	1.57 × 10^6^	3%
Ag-NP2 #4	4.50 × 10^−8^	2.22 × 10^7^	36%
Ag-MOD #2	3.11 × 10^−6^	2.97 × 10^5^	0.5%
**Line and Spot laser for large scale sintering**			
Ag-NP1, Spot Laser	3.05 × 10^−7^	3.28 × 10^6^	5%
Ag-NP1, Line Laser	4.85 × 10^−7^	2.06 × 10^6^	3%
Ag-NP2, Line Laser	1.35 × 10^−7^	7.41 × 10^6^	12%

## Data Availability

Data are contained within the article.
